# An Unusual Cause of Refractory Hypothyroidism

**DOI:** 10.7759/cureus.23522

**Published:** 2022-03-26

**Authors:** Omead J Mirgoli, Vishal Ramjas, Samhitha Munugoti, Heather Silverstein, Fawad Malik, Ahmed Salem, Frank Cassavell, Adam Atoot

**Affiliations:** 1 Family Medicine, Hackensack Meridian Health Palisades Medical Center, North Bergen, USA; 2 Medicine, California Institute of Behavioral Neurosciences and Psychology, Fairfield, USA; 3 Internal Medicine, Prime Health Care Consortium at St. Mary's and St. Clare Denville Hospital Program, Denville, USA; 4 Internal Medicine, Nova Southeastern University Dr. Kiran C. Patel College Of Osteopathic Medicine, Fort Lauderdale, USA; 5 Pharmacy, Bellevue Hospital, New York City, USA; 6 Internal Medicine, Hackensack Meridian Health Palisades Medical Center, North Bergen, USA; 7 Internal Medicine, Hackensack Meridian School of Medicine, Nutley, USA

**Keywords:** thyroid-stimulating hormone (tsh), malabsorption, levothyroxine, h pylori infection, refractory hypothyroidism

## Abstract

Refractory hypothyroidism has been increasingly identified worldwide. Primary hypothyroidism is considered refractory when there is a persistent elevation of thyroid-stimulating hormone (TSH) above the upper limit of normal despite escalating doses of levothyroxine with or without the persistence of hypothyroid symptoms. Further escalation of levothyroxine to supratherapeutic doses could be associated with potential complications such as iatrogenic hyperthyroidism, cardiac failure, and other conditions. Therefore, physicians should rule out non-compliance and pursue a further evaluation to identify etiologies for increased requirements or decreased absorption of levothyroxine in patients not achieving therapeutic doses. Here, we present a 40-year-old Indian male with worsening refractory hypothyroidism that resolved following eradication of his *Helicobacter pylori* (*H. pylori*) infection. Herein, we highlight a unique and reversible cause of refractory hypothyroidism. With this case report, we hope to encourage physicians to include *H. pylori* testing in the evaluation of primary hypothyroidism refractory to treatment.

## Introduction

The mainstay treatment of primary hypothyroidism is hormone replacement therapy. Levothyroxine, a synthetic form of thyroxine (T4), is generally considered a lifelong treatment among patients who have been diagnosed with hypothyroidism. With a narrow therapeutic index, many factors can interfere and alter the therapeutic efficacy of this treatment. A significant number of patients fail to respond to adequate doses of T4. Malabsorptive conditions can hinder or inhibit gastrointestinal uptake of oral levothyroxine and are an emerging cause of refractory hypothyroidism. Infection with *Helicobacter pylori* (*H. pylori*), a common bacteria affecting the stomach that is highly prevalent in developing countries [1], can cause a malabsorptive state where T4 uptake is reduced [2]. Herein, we present a case of a 40-year-old male with refractory hypothyroidism who was found to have malabsorption secondary to *H. pylori* infection. Following the treatment and eradication of his infection, there was significant improvement and normalization of the patient's thyroid-stimulating hormone (TSH). This case report aimed to encourage clinicians to include *H. pylori* in the differential diagnosis of refractory hypothyroidism.

## Case presentation

A 40-year-old Indian male with a past medical history significant for hypothyroidism, dyslipidemia, and pernicious anemia presented to the clinic for his annual physical examination. The patient reported a history of hypothyroidism for which he had been taking levothyroxine 100 mcg every morning. He denied fatigue, cold intolerance, hair loss, constipation, or weight gain. The patient denied any dietary restrictions and noted that he occasionally ate meat and eggs. The patient has no history of hospitalizations, and his family history was noncontributory. 

On evaluation, laboratory results revealed an elevated thyroid-stimulating hormone of 6.77 uIU/mL, positive thyroperoxidase antibodies, and positive thyroglobulin antibodies. Lipid studies also indicated an low-density lipoprotein (LDL) of 141 mg/dL (ref. range: 0-99 mg/dL), total cholesterol of 210 mg/dL (ref. range: 100-199 mg/dL), and triglycerides of 161 mg/dL (ref. range: 0-149 mg/dL). Other notable laboratory findings were an elevated serum aspartate aminotransferase (AST) and alanine aminotransferase (ALT) of 69 IU/L and 103 IU/L, respectively, which improved in subsequent testing. The remainder of the laboratory evaluation, including complete blood count and urinalysis, was unremarkable. There were no appreciable significant physical examination findings. The patient was lost to follow-up, and there were no changes in the medical management at that time.

During a subsequent follow-up visit six months later, laboratory results indicated a worsening TSH of 8.58 uIU/mL, total cholesterol of 220 mg/dL, and triglyceride level of 223 mg/dL. The patient’s dose of levothyroxine was increased from 100 mcg to 125 mcg, and repeat lab work was ordered six weeks later. The patient was lost to follow-up and was not seen until one year later. Lab work during this visit showed a continued worsening TSH value of 65.90 uIU/mL and a vitamin B12 level of 323 pg/mL (Table 1).

**Table 1:** Thyroid function testing and vitamin B12 levels T3: triiodothyronine; T4: thyroxine; TSH: thyroid-stimulating hormone; Ab: antibody; TPO: thyroid peroxidase

	Reference range	December 20, 2021	October 31, 2021	October 26, 2021	October 11, 2019	April 20, 2019
T3 uptake (%)	24-39	-	-	15	-	-
T3, total (ng/dL)	71-180	-	-	81	127	129
T4, free (ng/dL)	0.82-1.77	-	-	0.70	1.16	1.37
TSH (uIU/mL)	0.450-4.50	4.040	54.900	65.900	8.580	6.770
TSH receptor Ab (IU/L)	0.00-1.75	-	-	-	-	<0.50
Thyroglobulin Ab (IU/mL)	0.0-0.9	-	-	-	-	28.3
TPO Ab (IU/mL)	0-34	-	-	-	-	310
Vitamin B12 (pg/mL)	232-1245	>2000	342	323	-	202

**Figure 1:**
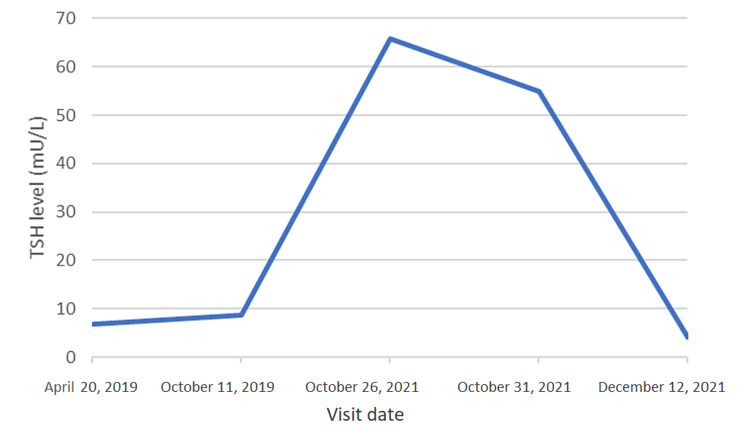
Patient’s TSH levels at each of his past five visits (the patient was treated for Helicobacter pylori on October 31, 2021) TSH: thyroid-stimulating hormone

**Figure 2:**
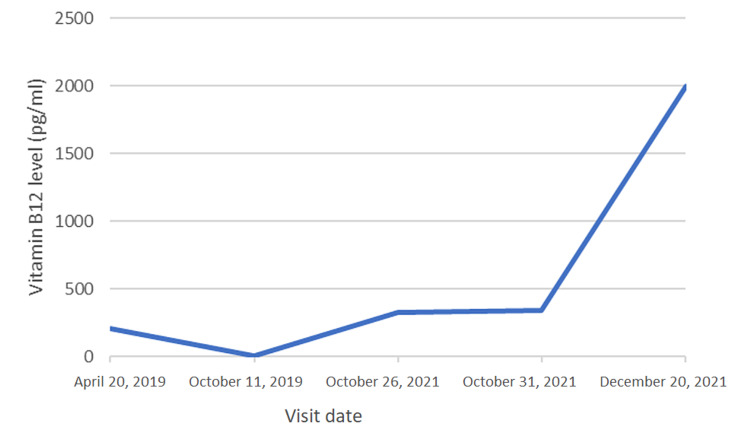
Patient’s vitamin B12 levels at each of his past five visits (the patient was treated for Helicobacter pylori on October 31, 2021)

Due to these findings, a possible malabsorptive etiology was suspected, and *H. pylori* breath test, celiac disease panel, and pernicious anemia panel were ordered. Results demonstrated negative antiparietal cell and intrinsic factor antibodies; however, *H. pylori* urea breath testing was noted to be positive. The patient was subsequently started on a 14-day regimen of triple therapy (amoxicillin, clarithromycin, and pantoprazole) for eradication of *H. pylori* infection. Levothyroxine was increased to 150 mcg at this time. Repeat *H. pylori* urea breath testing confirmed eradication of bacteria. After confirmed eradication, lab work from two weeks later demonstrated an improved TSH level of 4.04 uIU/mL and vitamin B12 level of >2000 pg/mL (Figures 1, 2).

## Discussion

Primary hypothyroidism is a condition caused by the destruction of the thyroid gland, leading to decreased production of triiodothyronine (T3) and thyroxine (T4). Diagnosis and the appropriate management of this condition are essential in preventing potential sequela [2]. Levothyroxine is the mainstay treatment in the majority of cases in which a gradual titrating dose is given to reach an optimal euthyroid state [2].

The preferred route of administration of levothyroxine is in its oral form. Upon ingestion, the majority of its absorption occurs within the small intestine, particularly in the jejunum and ileum [3,4]. A vital prerequisite for intestinal absorption includes a “gastric phase,” which consists of disintegration, dissolution, and precipitation of the drug [5]. The gastric acid during this phase contributes to the release of the active component of the drug from its ingested form, thus facilitating intestinal absorption [5]. Patients are usually started on an initial dose of levothyroxine of 1.6 mcg/kg body weight/day. However, changes in the initial dosage may have to be considered based on the patient’s age, comorbid conditions such as coronary heart disease, or factors altering the absorption of levothyroxine [6].

Refractory hypothyroidism has been increasingly identified worldwide. Primary hypothyroidism is considered refractory when there is a persistent elevation of thyroid-stimulating hormone (TSH) above the upper limit of normal despite escalating doses of levothyroxine with or without the persistence of hypothyroid symptoms. Although no definitive criteria exist, the typical levothyroxine dosage beyond which hypothyroidism is considered refractory is 1.9 ug/kg daily [7-9]. Further escalation of levothyroxine to supratherapeutic doses could be associated with potential complications such as iatrogenic hyperthyroidism and cardiac failure. Therefore, physicians should rule out non-compliance and pursue a further evaluation to identify etiologies for increased requirements or decreased absorption of levothyroxine (Table 2) [10-12].

**Table 2:** Common causes of refractory hypothyroidism

Non-compliance to levothyroxine	
Increased requirements of levothyroxine	Pregnancy weight gain
Increased clearance of levothyroxine	Phenobarbital, phenytoin, carbamazepine, rifampicin
Malabsorption of levothyroxine	Dietary: coffee, papaya, grapes, soy, herbal remedies, dietary fiber. Drugs: proton pump inhibitors, sucralfate, bile acid sequestering agents, ferrous sulfate, calcium carbonate. Diseases: autoimmune atrophic gastritis, *Helicobacter pylori* infection, small intestinal bacterial overgrowth, celiac disease, short bowel syndrome, lactose intolerance

After confirming adequate compliance in our patient, the levothyroxine dosage was increased to 125 mcg/day. As shown in Table 1, follow-up labs revealed worsening TSH levels despite the increased levothyroxine dose. At this juncture, refractory hypothyroidism was considered. The patient’s history suggested no etiologies for increased requirements or clearance of levothyroxine. In addition, further workup also revealed low vitamin B12 levels triggering a suspicion for a malabsorptive state causing a reduction in levothyroxine absorption. The patient’s history had no dietary restrictions nor drug interactions leading to malabsorption. Therefore, urea breath test, transglutaminase antibodies, antiparietal cell antibodies, and intrinsic factor antibodies were ordered to rule out *H. pylori* infection, celiac disease, and autoimmune atrophic gastritis, respectively. Laboratory results were negative for antibodies, and urea breath test was positive, confirming the presence of *H. pylori* infection. 

Infection due to *H. pylori* initially causes gastritis involving the superficial layer of the antrum mucosa. During this phase, there is an inflammatory mononuclear and plasma cell infiltration and increased gastrin levels leading to increased gastric acid production. Worsening gastritis can lead to atrophic pangastritis and intestinal metaplasia, affecting gastrin production leading to hypo or achlorhydria [13-16]. In addition, *H. pylori* neutralizes the existing gastric acid due to urease production, resulting in a hypo or achlorhydria state [11]. The hypo or achlorhydria affects the “gastric phase” of levothyroxine absorption, causing less release of the active ingredient from its ingested form.

Our patient was started on an *H. pylori* triple therapy regimen with amoxicillin, clarithromycin, and pantoprazole for 14 days. After completion of therapy and two-week abstinence from pantoprazole, a repeat urease breath test was negative, confirming the eradication of the *H. pylori* infection. A repeat thyroid function panel two weeks after confirming eradication of *H. pylori* infection showed normalization of TSH and vitamin B12 levels. These findings suggest that the *H. pylori* infection played a significant role in the treatment refractoriness of hypothyroidism.

## Conclusions

Refractory hypothyroidism can be caused by a malabsorption syndrome, which is commonly associated with *H. pylori* infection. A systematic approach to rule out possible causes in a patient’s failure to achieve a euthyroid state with escalating doses of levothyroxine is essential to prevent adverse effects and avoid unneeded testing and cost. This case is presented to emphasize the connection between *H. pylori* infection, malabsorption, and treatment failure with supratherapeutic levothyroxine doses. We aimed to provide insight into the growing literature surrounding *H. pylori* infection and refractory hypothyroidism. Thus, we advocate for clinicians to consider this relationship in evaluating patients presenting with refractory hypothyroidism without evident causes.
